# Clinical significance of NT-proBNP as a predictive biomarker of depressive symptoms in cardiac patients

**DOI:** 10.3389/fcvm.2025.1439520

**Published:** 2025-04-01

**Authors:** Syeda Humayra, Noorazrul Yahya, Chai Jia Ning, Mohd Asyiq Al-Fard bin Mohd Raffali, Imtiyaz Ali Mir, Abdul Latiff Mohamed, Hanani Abdul Manan

**Affiliations:** ^1^ Makmal Pemprosesan Imej Kefungsian (Functional Image Processing Laboratory), Department of Radiology, University Kebangsaan Malaysia, Kuala Lumpur, Malaysia; ^2^ Diagnostic Imaging & Radiotherapy Program, School of Diagnostic & Applied Health Sciences, Faculty of Health Sciences, Universiti Kebangsaan Malaysia, Kuala Lumpur, Malaysia; ^3^ Department of Radiology and Intervention, Hospital Pakar Kanak-Kanak (UKM Specialist Children’s Hospital), Universiti Kebangsaan Malaysia, Kuala Lumpur, Malaysia; ^4^ Cardiology Unit, Department of Medicine, Hospital Canselor Tuanku Muhriz, Universiti Kebangsaan Malaysia, Kuala Lumpur, Malaysia; ^5^ Department of Physiotherapy, M Kandiah Faculty of Medicine and Health Sciences, Universiti Tunku Abdul Rahman, Kajang, Malaysia; ^6^ Faculty of Medicine, University of Cyberjaya, Cyberjaya, Malaysia

**Keywords:** CVD, depression, heart failure, NT-proBNP, clinical prognosis

## Abstract

**Introduction:**

Depression is a significant comorbidity linked to poor subjective and objective health outcomes in cardiac patients. The paucity of data necessitates further research to elucidate the pathophysiological connection between depression and cardiac diseases in the presence of N-terminal pro-brain natriuretic peptide (NT-proBNP). Therefore, the current systematic review investigated the clinical significance of NT-proBNP as a predictive biomarker in cardiac patients with depressive symptoms.

**Methods:**

Two researchers independently performed an extensive search of published literature from inception until April 2024 on PubMed, Web of Science, and Cochrane Library in accordance with the PRISMA guidelines. From a total of 452 records, 29 articles were eligible for full-text review, whereof, data from 14 articles were systematically collated. Based on the Newcastle-Ottawa Scale (NOS) criteria, all studies earned 6–9 stars and were of good quality.

**Results:**

Among a total population of 4,035, male patients were predominantly higher (*n* = 2,618, 65.0%). Approximately, 31.3% (*n* = 1,264) cardiac patients were depressed. The mean age ranged between 56 and 76 and 58–73 years for depressed vs. non-depressed individuals respectively. More than half of the patients presented with heart failure (*n* = 2,234, 55.4%), followed by acute myocardial infarction (*n* = 1,368, 34.0%), coronary artery disease (*n* = 674, 16.7%), and acute coronary syndrome (*n* = 164, 4.1%). Poor ventricular function (26.1 ± 6.8 to 37.65 ± 12.71) and worsened NYHA class II-III functional symptoms (moderate-marked limitations) were more prevalent in depression. In addition, three studies found that age and female gender were significant risk factors in depressed patients. Significant clinical relevance was established between increased NT-proBNP and depressive symptoms in seven studies. NT-proBNP values ranged between 138 and 12,000 pg/ml vs. 108 to 6,000 pg/ml for depressed vs. non-depressed patients.

**Conclusion:**

The presence of elevated NT-proBNP in depression demonstrated adverse cardiovascular outcomes and played a crucial role in predicting the clinical prognosis. Future NT-proBNP studies with predefined follow-up period at different time intervals, and in clinically depressed patients are highly recommended.

**Systematic Review Registration:**

https://www.crd.york.ac.uk/PROSPERO/, identifier (CRD42024536115).

## 1 Introduction

Depression is a severe mental ailment affecting a substantial proportion of the global population (1). It often coexists with cardiovascular diseases (CVDs), a phenomenon commonly observed in clinical practice due to the frequent overlap between psychiatric and cardiac conditions (2). Both depression and ischemic heart disease (IHD) significantly influence annual morbidity and mortality rates, with depressed cardiac patients showing a 33% higher risk of death compared to those without depression (3). Epidemiological studies have validated the significant coexistence of these two conditions, and the mechanisms underlying their bidirectional relationship are complex and multifactorial (1). Specifically, depression is a strong predictor of CVD development and is closely linked to poor cardiovascular health and mortality (2). Nearly 45% of patients with acute myocardial infarction (AMI) exhibit depressive symptoms, and this co-occurrence is associated with poorer prognosis, as only 10% of these patients receive adequate treatment (3). Similarly, individuals diagnosed with coronary artery disease (CAD) may also experience elevated levels of psychological distress, particularly depressive symptoms, which contribute to a higher risk of adverse cardiac events (4, 5). Poor health behaviours and the pathophysiological correlates of depression, including coagulation, autonomic cardiovascular control, and the activation of neuroendocrine and inflammatory processes, most likely mediate the adverse outcomes (4). Depression is also highly prevalent among hospitalized patients with heart failure (HF) in the advanced stages of CVD, with 46.83% reported to exhibit depressive symptoms (6). A previous study from China (7) indicated that depression is a poor predictor of chronic heart failure (CHF) trajectory and outcome. In addition, hospitalizations and other episodes of CHF further exacerbate depression symptoms, resulting in an increased mortality rate and healthcare utilization (8). Consequently, the European Society of Cardiology advises an early screening for major depression, and in case of depressed cardiac patients, a combination of pharmaceutical treatment, psychosocial therapies, and cognitive behavioural therapy is highly recommended (8). Additionally, a recent scientific statement by the American Heart Association emphasizes that even mild depressive symptoms in patients with acute coronary syndrome (ACS) can result in elevated cardiac events (9). It has been proposed by experts that understanding the inflammatory responses involved in depression and HF could lead to effective therapeutic approaches (10). Although high-sensitivity C-reactive protein (hsCRP) was studied as an independent marker of depression severity in HF (11), nevertheless, the involvement of plasma N-terminal pro-brain natriuretic peptide (NT-proBNP) remains understudied (10).

HF and cardiac dysfunction affect about 26 million people globally and are major public health issues. With the rapid aging of the population, the burden of HF and heart dysfunction worldwide is escalating more significantly (12). Brain natriuretic peptide (BNP) and N-terminal proBNP (NT-proBNP) are commonly adopted clinical biomarkers for diagnosing HF and cardiac dysfunction (12). NT-proBNP is a kind of neurohormone mostly generated and released by the ventricular myocardium in response to myocardial wall stress and cardiac volume overload. In individuals with AMI, this biological marker has been found to be an important predictor of death and future MI attack (13). BNPs are accountable for regulating blood pressure and volume, often found in higher concentrations in patients with CAD. Studies on heart patients have recently linked atrial natriuretic peptide (ANP) to decreased anxiety; however, there is insufficient data regarding BNPs', specifically NT-proBNPs' impact on various aspects of wellbeing and mental health including depression (14). Although NT-proBNPs serve as critical indicators of the severity of cardiac illness, their significance in mental health remains unclear and demands further exploration (14). Scientific evidence revealed elevated NT-proBNP levels among patients with major depressive disorder (MDD) in the absence of CVDs, and the findings implicate a possible correlation between NT-proBNP levels and depressive symptoms (15). However, the results remained inconclusive since some researchers were unable to establish a statistically significant correlation between NT-proBNP levels and depression among stable CAD patients with MDD (16).

In cardiac patients, depression is a common comorbidity that is linked to unfavourable subjective and objective health outcomes (9). Examining the connection between depressive symptoms and clinical biomarker levels is especially important since depressive symptoms are highly prevalent in the aforementioned cardiac conditions (13). Since depression may be a modifiable risk factor for improving prognostic outcomes in CHF patients, addressing it is crucial for their clinical treatment and long-term prognosis (6, 17). Research has shown that when HF patients demonstrated improvements in depression, their possibility of death decreased dramatically (17). Thus, in clinical practice, it is critically vital to manage depression in these patients (17). The paucity of data in current literature about depression management and treatment in cardiac patients highlights the need for more research to elucidate the pathophysiological connection between depression and cardiac disease in the presence of NT-proBNP biomarker. Exploring the co-morbid status may be beneficial in comprehending the etiopathological mechanisms underlying depression in cardiac illnesses and promote supportive/preventive therapeutic guidelines for patients. Therefore, the present review systematically and critically aims to investigate the clinical significance of N-Terminal Pro-Brain Natriuretic Peptide (NT-proBNP) as a predictive biomarker in cardiac patients with depressive symptoms.

## 2 Methods

A systematic review of literature was conducted to investigate the clinical significance of NT-proBNP in depressed cardiac patients. Two researchers independently performed an extensive search of published literature from inception until April 2024 on PubMed, Web of Science, and Cochrane Library in accordance with the Preferred Reporting Items for Systematic Reviews and Meta-Analysis (PRISMA) (18) and followed past study guidelines (19–23). The search was restricted to English language, human studies, and original research articles only. To identify potentially relevant articles, the authors used key search terms “N-terminal pro b-type natriuretic peptide”, “NT-proBNP”, “depressive symptoms”, and “depression”. A manual search of the included articles' reference lists was also carried out independently by two authors. The review protocol was registered in the International Prospective Register of Systematic Reviews database (CRD42024536115).

**Figure 1:**
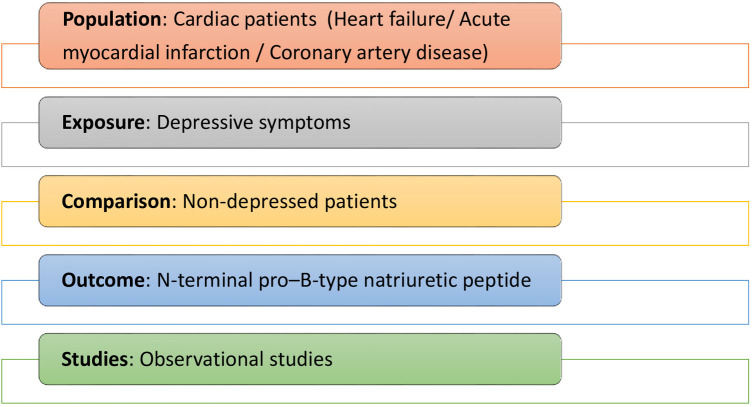
PICOS criteria for studies inclusion.

After the exclusion of duplicate files, two reviewers evaluated the eligibility criteria of potential studies and independently screened the titles and abstracts for inclusion. The full-text records of these studies were considered eligible if they aligned with the pre-specified PECOS model as shown in Figure 1. Inclusion criteria included: (i) observational studies; (ii) having measurements of depressive symptoms and NT-proBNP (mean/median) values; (iii) with cardiac subjects (HF/CAD/AMI) exposed to depression; (iv) compared to control patients without depression. Exclusion criteria included non-cardiac populations, studies with other psychometric measures (e.g., anxiety/stress), missing outcome measures, and unpublished results/preprints/conference proceedings. Papers in which the association between presence/absence of depression and NT-proBNP was unclear/missing or could not be identified were also excluded. Finally, two reviewers independently reached a mutual consensus to include or exclude studies, and disagreements between them were resolved by a third reviewer/coauthor. In addition, the quality of the included studies was assessed using the Newcastle-Ottawa Scale (NOS), and a study earning ≥ 6 stars was defined as good/high quality study with lesser risk of selection bias (24). Any disagreements in scoring the study quality based on the NOS checklist/criteria were also resolved through mutual consensus.

All relevant data from the included articles was extracted independently by two reviewers using a structured excel sheet table which was then verified by a third reviewer for final validation. The general attributes of the studies were collected, such as the name of first author, year of publication, location where the study was conducted, study design and population, total number of study participants, follow-up period, psychometric tool and the cut-off scores for depression. The participant characteristics such as age, gender, New York Heart Association (NYHA) classification, left ventricular ejection fraction (LVEF), CVD category, prevalence of depressive symptoms, antidepressant therapy, and adverse clinical events were recorded. In addition, the NT-proBNP values and depression scores were also determined to quantify and interpret the clinical significance of NT-proBNP in cardiac patients with depressive symptoms. Lastly, all extracted data were rechecked and disagreements were resolved by consensus. Due to different cardiac patient populations and outcome measures, a meta-analysis was not feasible for this study. The *P*-value for inferring statistical significance, mean value with standard deviation, median value with interquartile range, NT-proBNP values and depression scores were extracted and tabulated to qualitatively summarize the authors' findings.

## 3 Results

### 3.1 Screening results

**Figure 2:**
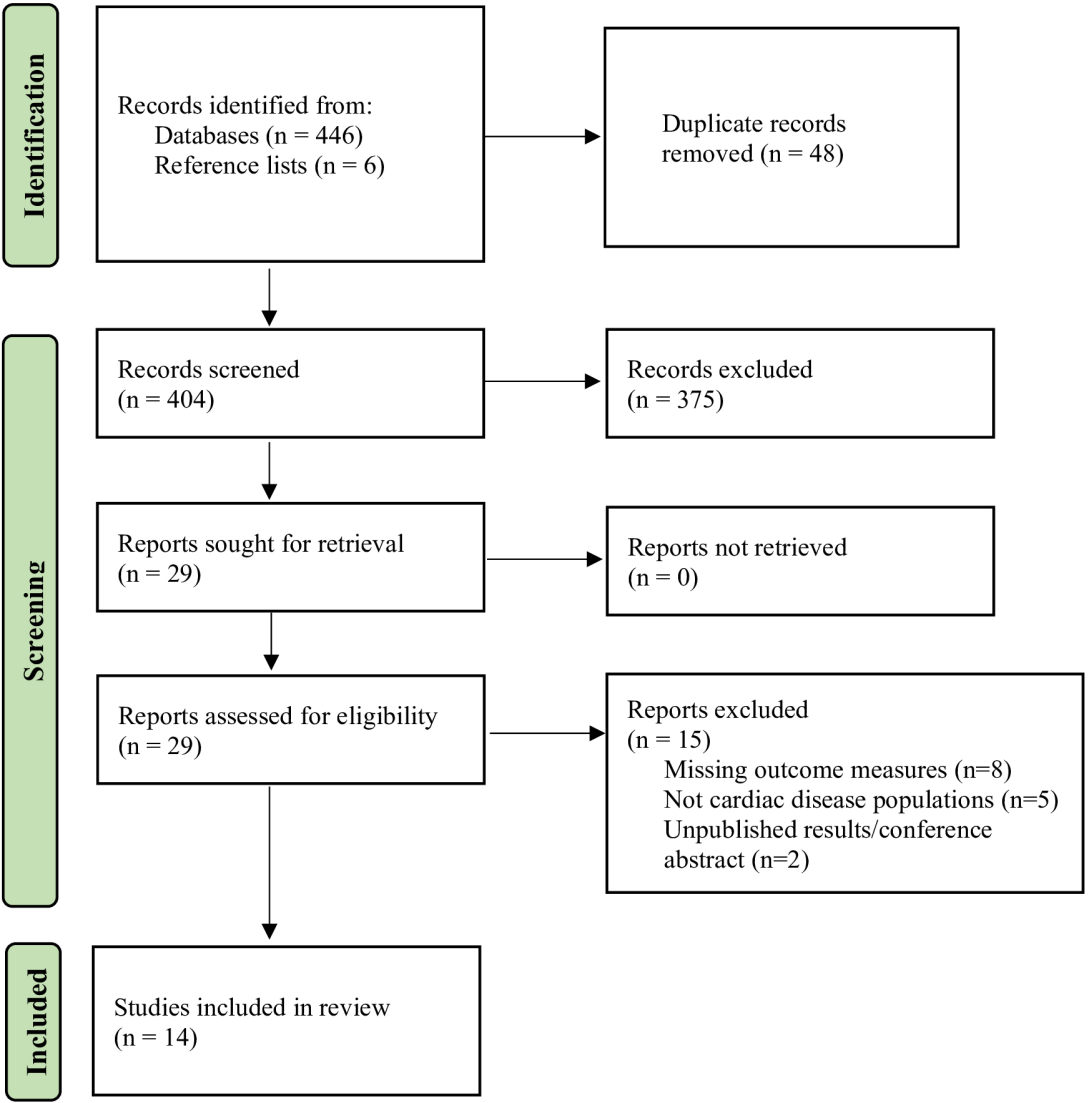
PRISMA flowchart of study selection process.

446 articles were produced in the initial search, and additional 6 papers were identified on manual search of the reference lists. After screening the titles and abstracts, 29 articles were considered eligible for a full-text review. Finally, 14 articles were systematically reviewed and the findings were qualitatively reported. The PRISMA flowchart is presented in Figure 2.

### 3.2 Study characteristics and quality

The included studies were either conducted in a single cohort presenting cardiac patients with depressive symptoms (7, 25) or alongside a comparator cohort involving cardiac patients without depressive symptoms (6, 26–30). The other six studies were performed in a cross-sectional setting, whereby four studies compared the study outcomes in depressed vs. non-depressed individuals (13, 16, 31, 32), and the remaining two studies had no comparator groups (14, 33). Majority of the studies included patients with HF/CHF (*n* = 8 studies) and CAD/CHD (*n* = 3 studies), followed by AMI (*n* = 2 studies) and ACS (*n* = 1 study). The sample size ranged from 72 to 1,265 subjects. The minimum follow-up duration was one month (31) and the maximum was 5.8 years (28). In terms of psychometric assessment, majority of the included studies utilized the Patient health questionnaire (PHQ-9) (6, 7, 14, 25, 31–33) and the Hospital Anxiety and Depression Scale (HADS) for measuring the symptoms/severity of depression (26, 27, 30). Out of all, only one study (16) clinically diagnosed depressed patients using the Structured Clinical Interview for Diagnostic and Statistical Manual of Mental Disorders 4th edition (SCID- IV), while others mostly used reliable self-reported tools for assessing depression. In addition, one study (29) categorized the patients based on the Center for Epidemiologic Studies Depression Scale (CES-D) score quintiles (0–1, 2, 3–4, 5–8, and ≥9), whereas the other study (31) evaluated the depressive symptoms based on highest vs. lowest PHQ score quartile and cognitive vs. somatic types. The cut-off values differed depending on the kind of tool and the depression scoring criteria, however, in most papers (6, 7, 14, 32, 33), a cut-off point above 10 was widely used for PHQ-9. Regionally, majority of the studies were performed in North America (*n* = 7 studies) (16, 27–30, 32, 33), Europe (*n* = 5 studies) (7, 14, 25, 26, 31), and East Asia (*n* = 2 studies) (6, 13). The publication time frame ranged from 2006 to 2022. All studies earned 6–9 stars and were defined as good/high-quality studies according to the NOS assessment criteria. The study characteristics are tabulated in Table 1.

**Table 1:** Study characteristics.

First author, Year	Country	Study design	Study population	Study groups	Sample Size	Follow-up	Psychometric tool (cutoff score)	Depression diagnostic criteria	NOS quality score
Duan et al. (6)	China	Cohort	HF	Depressed (*n* = 123)/non-depressed (*n* = 141)	264	2 years	PHQ-9 (≥10)	Self-reported	9✯
Müller-Tasch et al. (7)	Germany	Cohort	CHF	None	180	1 year	PHQ-9 (≥10)	Self-reported	8✯
Lossnitzer et al. (25)	Germany	Cohort	CHF	None	446	2 years	PHQ-9 (≥9)	Self-reported	8✯
Vu et al. (29)	USA	Cohort	HF	CES-D score quartiles	303	5.5 years	CES-D (≥9)	Self-reported	9✯
Zuzarte et al. (30)	Canada	Cohort	CHF	Depressed (*n* = 60)/non-depressed (*n* = 64)	124	1 year	HADS (≥7)	NR	8✯
Fangauf et al. (14)	Germany	Cross-sectional	CAD	None	529	No follow-up	PHQ-9 (≥10)	Self-reported	8✯
Celano et al. (33)	USA	Cross-sectional	ACS	None	164	6 months	PHQ-9 (≥10)	Self-reported	7✯
Ren et al. (13)	China	Cross-sectional	AMI	Depressed (*n* = 36)/non-depressed (*n* = 67)	103	No follow-up	SDS (≥50)	Self-reported	7✯
Brouwers et al. (26)	Denmark	Cohort	HF	Depressed (*n* = 16)/non-depressed (*n* = 78)	94	9 months	HADS-D (≥8) BDI (≥10)	Self-reported	7✯
Smolderen et al. (31)	Netherlands	Cross-sectional	AMI	Depressed (Somatic: *n* = 355; Cognitive: *n* = 382)/non-depressed (Somatic: *n* = 910; Cognitive: *n* = 883)	1,265	1 month	PHQ-9 [Somatic depressive symptoms (≥4)] [Cognitive depressive symptoms ≥2)]	Self-reported	8✯
Van den Broek et al. (28)	USA	Cohort	HF	Depressed (*n* = 75)/non-depressed (*n* = 133)	208	5.8 years	CES-D (≥8)	NR	8✯
Song et al. (32)	USA	Cross-sectional	HF	Depressed (*n* = 57)/non-depressed (*n* = 153)	210	1 year	PHQ-9 (≥10)	NR	7✯
Bankier et al. (16)	USA	Cross-sectional	CHD	Depressed (*n* = 30)/non-depressed (*n* = 42)	72	No follow up	GAF (≤70)	Clinically diagnosed MDD (SCID- IV)	6✯
Bunevicius et al. (27)	USA	Cohort	CAD	Depressed (*n* = 7)/non-depressed (*n* = 66)	73	No follow up	HADS (≥10)	Self-reported	7✯

ACS, acute coronary syndrome; AMI, acute myocardial infarction; BDI, beck depression inventory; CAD, coronary artery disease; CES-D, center for epidemiologic studies depression scale; CHD, coronary heart disease; CHF, chronic heart failure; GAF, global assessment of functioning scale; HADS-D, hospital anxiety and depression scale—depression; HAMD, hamilton rating scale for depression; MDD, major depressive disorder; NOS, newcastle-ottawa scale; PHQ, patient health questionnaire; SCID, structured clinical interview for diagnostic and statistical manual of mental disorders fourth edition; SDS, zung self-rating depression scale.

### 3.3 Demographic and clinical characteristics

Among a total population of 4,035, male patients were predominantly higher than females (*n* = 2,618, 65.0% vs. *n* = 1,417, 35.1%). Approximately, 31.3% (*n* = 1,264) of the cardiac patients presented with depressive symptoms. The mean age ranged from 56 ± 9 to 76 ± 9 vs. 58 ± 11 to 73 ± 12 for depressed vs. non-depressed patients respectively. It was noted that younger age (6, 29, 32) and female gender (28, 29, 31) were significant risk factors among patients with depression compared to those without depression. In addition, more than half of the patients presented with HF (*n* = 2,234, 55.4%), while others suffered from AMI (*n* = 1,368, 34.0%), CAD (*n* = 674, 16.7%), and ACS (*n* = 164, 4.1%). Most of the patients were in stage II-III of the NYHA functional classification and had reduced LVEF ranging from 26.1% ± 6.8 to 48.97 ± 11.09. Ischaemic cases were reported in almost 64.3% of the studies. Out of all studies, only 8 studies recorded patients who were treated with antidepressant medication (*n* = 121, 9.7%). HF rehospitalization (*n* = 153, 12.1%) and death (*n* = 85, 6.7%) were observed mostly among depressed cardiac patients. The detailed participants' characteristics are presented in Table 2.

**Table 2:** Participants characteristics.

	Patient/comparator							
Study	Age	Gender	NYHA Class	LVEF	CVD type	Depressive symptoms	Antidepressants	Adverse events
Duan et al. (6)	71.5 ± 11.2/67.6 ± 12.7 (*P* = 0.006)	M = 78 (63.41%) 98 (69.50%)	NYHA III = 50 (40.7%)/70 (49.7%)	NR	CAD = 57 (46.3%)/73 (51.8%)	130 (46.8%)	NR	HF readmission = 80 (30.3%), All-cause mortality = 13 (4.92%)
Müller-Tasch et al. (7)	69.5 ± 9.8	M = 128 (71.1%)	NYHA II = 117 (65.0%)	36.6 ± 7.3	IHD = 87 (48.3%)	43 (23.9%)	10 (5.6%)	NR
Lossnitzer et al. (25)	58.9 ± 14.2	M = 328 (73.5%)	NYHA I—II	37.65 ± 12.71	NR	72 (16.0%)	NR	NR
Vu et al. (29)	75.3 ± 5.3 (*P* = 0.001)	F = 195 (68.0%)*	NR	65.6 ± 5.9	CHD = 25 (9.0%)	287 (6.0%)	NR	NR
Zuzarteet al. (30)	76.2 ± 9.10/73.0 ± 12.0	F = 31 (51.7%)/25 (39.0%)	NYHA II = 25 (41.7%)/32 (50.0%)	NR	IHF = 32 (53.3%)/35 (54.7%)	60 (48.5%)	11 (47.8)	HF readmission = 17 (28.3%), All-cause mortality = 3 (5.0%)
Fangauf et al. (14)	59.3 ± 9.4	M = 417 (78.8%)	NYHA II = 237 (44.8%)	56.7 ± 14.4	NR	27 (5.4%)	NR	NR
Celano et al. (33)	61.5 (10.6)	M = 137 (84.0%)	NR	NR	NR	17 (10.0%)	27 (16.0%)	NR
Ren et al. (13)	62.9 ± 15.0/61.2 ± 12.6	M = 27 (75.0)/54 (80.6%)	NYHA II = 13 (36.1%)/28 (41.8%)	48.97 ± 11.09/47.57 ± 10.62	NR	36 (35.0%)	NR	NR
Brouwers et al. (26)	62 ± 9	M = 75 (80.0%)	NR	26.1 ± 6.8	IHD = 39 (42.0%)	HADS-D: 16 (17.0%) BDI: 41 (46.6%)	8 (8.0%)	Rehospitalization = 20 (22.0%)
Smolderen et al. (31)	Somatic = 58.7 ± 11.6/60.1 ± 11.8 Cognitive = 58.2 ± 11.3/60.4 ± 11.9 (*P* = 0.03)	Somatic: F = 159 (44.8%) 251 (27.6%) Cognitive: 162 (42.4%)/248 (28.1%)*	NR	<40%	Somatic: CAD=120 (33.8%) Cognitive: 144 (37.7%)	Somatic = 355 (28.1%) Cognitive=382 (30.2%)	NR	NR
Van den Broek et al. (28)	74.9 ± 6.5/75.3 ± 5.9	F = 50 (66.7%)/56 (42.1%)*	NR	Reduced LVEF = 27 (36.0%)	CHD = 46 (61.3%)/84 (33.2%)	75 (36.1%)	5 (6.7%)	Depressed high NT-proBNP group showed the greatest risk of CVD death (*n* = 56) compared to the non-depressed low NT-proBNP group (*n* = 38)
Song et al. (32)	56 ± 9/58 ± 11 (*P* < 0.001)	M = 34 (60.0%)/113 (74.0%)	NYHA III-IV = 44 (77.2%)/71 (46.4%)	32 ± 16/34 ± 13	IHD = 26/92	57 (27.1%)	28 (49.1%)	13 patients (6.2%) died, 36 (17.1%) were hospitalized, and 9 (4.3%) had ED visits due to decompensated HF
Bankier et al. (16)	67 ± 11/68 ± 13	M = 24 (80.0%)/32 (76.0%)	NR	NR	NR	30 (41.7%)	15 (50.0%)	NR
Bunevicius et al. (27)	60 ± 9/57 ± 10	M = 4 (57.0%)/50 (76.0%)	NYHA II = 59 (81.0%)	NR	HF = 46 (63.0%)	7 (10.0%)	17 (23.3%)	NR

* Significance level (*P* < 0.05).

CAD, coronary artery disease; CHD, coronary heart disease; CVD, cardiovascular disease; F, female; HADS-D, hospital anxiety and depression scale—depression; HF, heart failure; IHD, ischemic heart disease; LVEF, left ventricular ejection fraction; M, male; NR, not reported; NYHA, New York heart association.

### 3.4 Clinical significance of Nt-proBNP in depressed cardiac patients

Six out of 14 studies discovered no significant correlations between depression and NT-proBNP (7, 16, 25, 26, 28, 31), and one study found that NT-proBNP was linked to better mental health in stable CAD patients with depression (14). Nevertheless, a statistically significant connection was deduced between increased NT-proBNP values and higher depressive symptoms across seven studies (6, 13, 27, 29, 30, 32, 33). The depression scores ranged from 2 to 63, while the NT-proBNP values ranged between 138 and 12,000 pg/ml for depressed patients and 108 to 6,000 pg/ml for non-depressed cardiac patients. The coexistence of elevated NT-pro BNP and depressive symptoms predicted the shortest cardiac event-free survival in HF patients (32). Furthermore, depressed HF patients with high NT-proBNP experienced the greatest risk of cardiovascular mortality compared to the non-depressed cohort with low NT-proBNP values (28). The clinical significance of NT-proBNP among cardiac patients with depressive symptoms have been highlighted in Table 3.

**Table 3:** Clinical significance of NT-proBNP in cardiac patients with depressive symptoms.

Study	Patient/comparator		Statistical significance	Authors findings
	Depression score	NT-proBNP		
Duan et al. (6)	5.30 (2.66, 10.54)	3850.00 (2250.00–9585.00)/2320.00 (1217.00–5330.00)	*P* < 0.001	NT-proBNP was significantly higher among patients with depressive symptoms
Müller-Tasch et al. (7)	7.0 ± 5.3	1815.6 ± 3144.8	*P* = 0.49	No significant associations between NT-proBNP and psychometric variables
Lossnitzer et al. (25)	4.78 ± 4.1	966 ± 1,545.1	*P* = 0.971	No significant correlations between depression and NT-proBNP
Vu et al. (29)	2.0 (1–4)	138 (59–266)/108 (58–210)	*P* = 0.001	Highest CES-D score was significantly associated with NT-proBNP
Zuzarte et al. (30)	≥7	12,000/6,000	*P* = 0.021	Higher levels of NT-proBNP associated with greater severity of depression
Fangauf et al. (14)	9.9 ± 5.4	188.2 ± 382.6	*P* = 0.028	NT-proBNP was associated with better mental health including depression
Celano et al. (33)	4.3 (4.4)	536.6 (1022.5)	*P* = 0.017	Increased depressive symptoms were associated with higher concentrations of the prognostic marker NT-proBNP
Ren et al. (13)	54.78 ± 4.57	1135.0 (131.5, 2474.0)/384.0 (133.0, 990.0)	*P* = 0.013	Patients with depressive symptoms had significantly higher NT-proBNP levels as compared to patients without depressive symptoms
Brouwers et al. (26)	HADS-D: 4.5 ± 4.0 BDI: 10.7 ± 9.5	HADS: 931 (338–3,232)/973 (390–2,015) BDI: 849 (290–3,027)/958 (431–1,745)	*P* = 0.27 *P* = 0.22	No significant relationship between the psychological markers (i.e., depressive symptoms score on both HADS-D & BDI) and cardiac biomarker (NT-proBNP)
Smolderen et al. (31)	Somatic: ≥4 Cognitive: ≥2	Somatic: 2,800/2,600 Cognitive: 2,600/2,500	*P* = 0.30 *P* = 0.76	There were no significant associations between either of the depression dimensions and NTproBNP
Van den Broek et al. (28)	≥8	496 (159–1,632)/520 (148–1,716)	*P* = 0.85	In patients with HF at baseline, the mean NT-proBNP levels did not significantly differ between those with and without depression
Song et al. (32)	≥10	>581	*P* = 0.001	The coexistence of elevated NT-pro BNP and depressive symptoms predicted the shortest cardiac event-free survival in HF patients
Bankier et al. (16)	63 ± 6/72 ± 8	446.7 (636.9)/581.38 (1007.102)	*P* > 0.05	In case of NT-proBNP levels, there were no statistically relevant differences between depressed vs. non-depressed CHD patients
Bunevicius et al. (27)	≥10	761 ± 446/564 ± 170	*P* = 0.02	CAD patients with depressive symptoms had higher NT-pro BNP concentrations

BDI, beck depression inventory; CAD, coronary artery disease; CES-D, center for epidemiologic studies depression scale; CHD, coronary heart disease; HADS-D, hospital anxiety and depression scale—depression; NT-proBNP, N-terminal pro–B-type natriuretic peptide.

## 4 Discussion

The coexisting prevalence of comorbid CVD and depression is substantial but often underdiagnosed, with poor prognosis, uncertain therapeutic target, and incompletely elucidated unique pathogenesis (2). Anxiety-reducing qualities of ANP have been previously reported in patients with HF (34). On the contrary, data supporting the potential associations between BNP or NT-proBNP and anxiety, depression, or quality of life remain scant and inconsistent (7). Therefore, this systematic review contributes to the growing body of research on the complex interplay between depressive symptoms, cardiac biomarkers, and cardiovascular outcomes in depressed cardiac patients. The authors report a significantly higher prevalence of depressive symptoms and increased NT-proBNP concentrations among cardiac patients mostly among those with heart failure.

HF is frequently diagnosed using natriuretic peptides (BNP/NT-proBNP), which also serve as outcome predictors for patients with both preserved and reduced ejection fraction (35). In response to wall stress and volume overload, the cardiac myocytes in the ventricles of CHF patients tend to secrete natriuretic peptides, which help in maintaining blood pressure and emotion regulation (7). Specifically, both BNP and NT-proBNP are utilized as indicators of the progression and prognosis of CHF and guiding standardized treatment; however, N-terminal peptides are more frequently measured for research and clinical diagnostics due to their longer half-lives (7).

Heart patients are more likely than the general population to experience depression, anxiety, and distress, which is probably related to both psychological adaptation and neurobiological processes (14). Depression can worsen cardiac function through a variety of mechanisms, including inflammatory responses, endothelial dysfunction, platelet hyperactivation, hyperfunction of the hypothalamic-pituitary-adrenal axis, and autonomic dysfunction (6). In addition, genetic, biological, psychosocial, and behavioural factors involving the central and autonomic nervous systems, the neuroendocrine, immune, and the vascular and hematologic systems may also be held accountable in this coexistence (1). Particularly, pathophysiologic elements affecting these systems included the loss of heart rate variability in depression as a result of the homeostatic imbalance between the parasympathetic and sympathetic nervous systems (1).

According to the study findings, it can be suggested that plasma NT-proBNP levels may serve as a predictive biomarker for depression severity. One of the included studies (13) found that high NT proBNP levels were significantly associated with depressive symptoms, particularly somatic, cognitive, and core depression. This correlation raises the possibility that NT pro-BNP levels in AMI patients may be impacted by the somatic, cognitive, and emotional components of depression (13). In addition, the coexistence of high NT-pro BNP and severe depressive symptoms predicted a shorter cardiac event-free survival in HF patients. Whereas, in the absence of depressive symptoms, the same results were non-significant. It was found that both depressive symptoms and NT-pro BNP were highly predictive of cardiac event-free survival (32). These findings coincide with a meta-analysis of 4,012 HF patients, where depression was a significant marker of all-cause mortality (36). This implies that one of the possible connections between depression and elevated risk of adverse cardiac events may be related to the high NT-proBNP in plasma. Nevertheless, the precise mechanisms underlying the abnormal elevation of NT-proBNP in depressed patients is still not well-established (15). Previous research has shown that women experience depression twice as frequently as males across different cultures (37). However, most of our study population were male patients. This could be related to the higher incidence of CVD among men, since gender-based differences exist in various conditions including CAD and HF (38).

Despite the clinical significance of NT-proBNP in depressed cardiac patients, it should be noted that several studies failed to establish a significant link between depression and NT-proBNP. A plausible explanation could be that majority of the study population were not diagnosed with clinical depression and tend to present with mild-moderate depressive symptoms, which were probably inadequate to cause alterations in the cardiac biomarker level (14). Additionally, the small sample sizes might have also contributed to the inability to establish a significant correlation between NT-proBNP levels and depression (16, 26). At the same time, relationship between depression and NT-proBNP may be potentially impacted by the existence or severity of HF. The possible variations in NT-proBNP levels due to depression may be negligible when compared to the elevated values resulting from HF pathophysiology. This may be particularly meaningful for systolic HF, as patients with reduced LVEF typically demonstrate higher BNP or NT-proBNP levels than patients with preserved LVEF, presumably because the latter have lower end-diastolic wall stress (7).

Certain limitations in this study needs to be considered. For instance, the cognitive functioning was not pre-assessed in most studies, so the patient's mental health status was not thoroughly examined or perhaps underreported. Depression scores and NT-proBNP concentrations were obtained at a one-time point, and limited data were available on the long-term follow-up or cross-validation of results. It could not be identified whether pre-existing myocardial dysfunction might have been responsible for the alterations in NT-proBNP levels or it may be a result of the pre-existing symptoms of depression since most study findings were derived from non-causal conclusions. Across the studies, depression was assessed using self-reported tools and not through psychiatric evaluation. However, in most cases, PHQ-9 questionnaire was administered which corresponds to one of the 9 DSM-IV diagnostic criteria for MDD and suspects clinically relevant depression on a score of 10 or higher (7). Certain patients were reported to have renal insufficiencies, thus adding another comorbid condition and influencing the degree/extent of depressive symptoms. The underrepresentation of women in this review is also a notable limitation, as age-adjusted analyses indicate that women may demonstrate comparable CVD incidence rates to men after a certain age, despite a delay in the median age of CVD onset (39). Simultaneously, the underrepresentation of men in mental health studies is also highlighted, as men are less likely to seek help for psychological issues, more prone to misdiagnosis, and subjected to gender biases by clinicians and researchers (40). Lastly, the antidepressant treatment was either unknown, unreported, or unprescribed in some studies, which does not give us a clear idea about the clinical management of depression.

Understanding the relationship between depressive symptoms and NT-pro-BNP levels can provide useful insights into the complex interplay between mental health and cardiac function. Consistent monitoring may help to identify patients at higher risk of adverse cardiovascular events and tailoring treatment strategies to address the psychological and physiological aspects of their condition. The current therapeutic approach combines cardiovascular preventive regimens with the inclusion of antidepressants (2). Coping strategies in HF patients with depressive symptoms have been previously emphasized, highlighting the importance of managing psychological aspects in treating heart diseases (41). Simultaneously, the implementation of telemonitoring in CHF patients with moderate depression may also provide a positive impact on their quality of life by improving their depressive symptoms (8). Additionally, culturally specific interventions tailored for vulnerable patient groups based on their gender and ethnic differences should also be in practice for better management. Future NT-proBNP studies with predefined follow-up period at different time intervals, and in subjects with confirmed clinical depression are highly recommended.

## 5 Conclusion

The current study emphasizes how crucial it is to consider the presence/severity of depression symptoms among cardiac patients in order to comprehend how they may impact or link with cardiovascular biomarkers such as NT proBNP. Overall, NT-proBNP serves as a biomarker that reflects the physiological response to cardiac stress and dysfunction in cardiac patients. The elevation of NT-proBNP levels in the presence of depressive symptoms may indicate a synergistic effect of depression and cardiac condition on neurohormonal activation, ultimately affecting the clinical manifestations and prognosis of CVD. The aetiology of depression-complicated CVD remains elusive and multifaceted; thus, additional fundamental research is necessary to validate the clinical significance and role of NT-proBNP biomarker.

In summary, it can be concluded that patients suffering from HF with deteriorated functional symptoms and ventricular impairment are most likely to experience increased depressive symptoms, and the coexistence of high NT-proBNP plays a crucial role in predicting the clinical prognosis of these patients.

## Data Availability

The original contributions presented in the study are included in the article/Supplementary Material, further inquiries can be directed to the corresponding author.
